# PW01-017 – Urine MMP-3 level as a biomarker for in FMF attack

**DOI:** 10.1186/1546-0096-11-S1-A70

**Published:** 2013-11-08

**Authors:** B Sozeri, N Dincel, E Yilmaz, S Mir

**Affiliations:** 1 Pediatric Rheumatology, Ege University, Izmir, Turkey; 2 Pediatric Nephrology, Ege University, Izmir, Turkey

## Introduction

Matrix metalloproteinase-3 (MMP-3) has been implicated in experimental and clinical models of human inflammatory conditions. Increased levels of MMPs have been shown in serum and other body fluids such as synovial fluid in inflammatory conditions including ankylosing spondylitis, rheumatoid arthritis and juvenile idiopathic arthritis (JIA). Familial Mediterranean fever (FMF) is an autosomal recessive, inherited, autoinflammatory disease characterized by recurrent, self-limited bouts of fever and localized inflammation, usually involving the peritoneum, pleura, joints or skin.

## Objectives

To investigate whether level of urine matrix metalloproteinase-3 (MMP-3) can serve as a biomarker for monitoring and predicting attack in patients with FMF in daily clinical practice.

## Methods

We studied 50 (28 females, 22 males) patients who diagnosed with FMF according to Tel Hashomer criteria and 32 healthy (21 females, 17 males) controls. We determine all FMF subjects both in attack period (FMF-AP) and attack free period (FMF-AFP) groups. Serum and urine samples were obtained within the first 6–24 h of the AP, and 10 days later after the attack (AFP). The serum samples were measured on the same day while urine samples were collected on ice and divided into aliquots and frozen immediately and stored at -80°C until ready for assay.

## Results

The mean age at onset of symptoms was 57, 26±33.5 months. The most common symptom seen during the attacks was: fever (n=40, 80%) abdominal pain (n=36, 72%), arthritis (n=20, 40%) and others (myalgia, erysipelas like lesion, vasculitis, etc.) (n=6, 12%). In the genotype distribution of patients, homozygous M694V mutation was seen mostly (n=14, 28%). During AP, urine MMP-3 levels of patients was higher as well as during AFP and controls (2,32±0,51 ng/mL, 0.89 ± 2,29 ng/mL and 1,24 ± 0,17 ng/mL, respectively, p=0.00). In attack period, urinary MMP levels were detected higher in patients with arthritis than others (p <0.05). In addition urinary MMP-3 levels were significantly higher in male compared to female patients (2, 29 ± 0,45 versus 2,24±0,57, respectively, p=0,00). The patients with M694V allele (n=29) had statistically significant high levels of urine MMP-3 levels than other patients (2.37±0,56 versus 1,99±,31, p=0,04,respectively) . Also, acute phase reactants (WBC, SAA, fibrinogen CRP, ESR) were higher in patients with M694V allele but no there were no statistically significant (p=0.89, 0.75, 0.86, 0.85, 0.7, respectively).

## Conclusion

In this study we have focused on the presence and patterns of appearance of MMPs in the urine of subjects with FMF, and in healthy age-matched subjects. We showed that inflammation-specific MMP patterns may provide clinicians with valuable information in patients with FMF.

## Disclosure of interest

None declared

